# Fuyuan Xingnao Decoction Promotes Angiogenesis Through the Rab1/AT1R Pathway in Diabetes Mellitus Complicated With Cerebral Infarction

**DOI:** 10.3389/fphar.2021.616165

**Published:** 2021-02-17

**Authors:** Dong Deng, Yao Qu, Lihua Sun, Liyang Jia, Jianhong Bu, Miaoqing Ye, Zhenyi Chen, Yun Geng, Shuang Zhou, Bangjiang Fang

**Affiliations:** ^1^ Department of Emergency Medicine, LongHua Hospital Shanghai University of Traditional Chinese Medicine, Shanghai, China; ^2^ Department of Internal Medicine, Shanghai Municipal Hospital of Traditional Chinese Medicine, Shanghai, China; ^3^ Department of Liver Disease, Shaanxi Provincial Hospital of Traditional Chinese Medicine, Xi’an, China; ^4^ Department of Cardiology, the Second Clinical Medical College, Henan University of Traditional Chinese Medicine, Zhengzhou, China; ^5^ Department of Acupuncture and Massage College, Shanghai University of Traditional Chinese Medicine, Shanghai, China

**Keywords:** fuyuan xingnao decoction, diabetes mellitus complicated with cerebral infarction, brain microvascular endothelial cells, angiogenesis, Rab1/AT1R pathway

## Abstract

Fuyuan Xingnao decoction (FYXN), a traditional Chinese formula comprised of seven herbs, has been utilized to treat diabetes mellitus complicated with cerebral infarction (DMCI) for years. Yet, its protective and regulatory mechanism is poorly understood. The aim of the study is to investigate the effects of FYXN on DMCI *in vitro* and *in vivo*, as well as its mechanism in angiogenesis. For *in vivo* experiments, FYXN was administered to DMCI rats with streptozotocin (STZ) injection-induced diabetes. Then middle cerebral artery occlusion (MCAO) was conducted and the cerebral cortex sections of the rats were obtained. The ultrastructure of cerebral microvessels and new vessel density of ischemic penumbra were evaluated by the transmission electron microscopy (TEM) assay and immunohistochemistry, respectively. Protein and mRNA expression levels of Rab1/AT1R in cortex were assayed by Western blotting and real-time fluorescence quantitative real-time polymerase chain reaction (RT-qPCR). *In vitro*, FYXN serum was produced in rats on the fourth day 2 h after the last FYXN administration. Green fluorescence was observed after transfection with lentivirus packaged Rab1-WT or siRNA for 24 h. The activity of brain microvascular endothelial cells (BMECs) treated with sera from these rats was tested by MTT assay and Transwell assays, respectively. The expression of AT1R on the cell membrane and endoplasmic reticulum of BMECs was evaluated by immunofluorescence staining. Protein expression levels of signaling molecules in the Rab1/AT1R pathways were also detected. Results showed that *in vivo*, FYXN treatment significantly intensified CD31 staining in the cortical areas and enhanced the mRNA and protein levels of AT1R, Ang II, Rab1a, Rab1b and VEGF expression in ischemic cerebral cortex tissues. *In vitro*, the expression levels of AT1R, Ang II, Rab1a, Rab1b and VEGF in the cerebral infarction model group were significantly higher than those in the control group, with further increases after administration of FYXN drug serum. FYXN promoted the proliferation and migration of BMECs by activating the Rab1/AT1R signaling pathway. In conclusion, FYXN exerts a protective effect against DMCI by promoting angiogenesis via the Rab1/AT1R pathway, which provides strong evidence for the therapeutic effect of FYXN on DMCI.

## Introduction

Diabetes mellitus (DM), a major risk factor for cerebral infarction (CI), can directly induce and/or aggravate cerebrovascular damage (Gjerde and Naess, 2014). In DM patients, the recurrence rate of acute cerebral infarction (ACI) is higher and its prognosis is worse (Chen, R. et al., 2016). Thus, investigating the pathogenesis is a critical step before devising an effective treatment strategy for DMCI.

Vascular damage is a major complication of DM. Apart from damaging blood vessels (Yao et al., 2019), diabetic hyperglycemia also induces substantial local ischemia. The insulin resistance enhances platelet adhesion, activation, and aggregation (Huang et al., 2017). The hemodynamic disorder impairs vascular wound healing, and reduces collateral circulation, leading to insufficient blood flow and then CI or ischemic stroke.

Fuyuan Xingnao decoction (FYXN) was produced based on the academic experience (Hu, 2005) of Professor Hu Jianhua, a famous doctor of Traditional Chinese Medicine (TCM) in Longhua Hospital, Shanghai University of TCM. FYXN presents a favorable clinical response, and its therapeutic targets and mechanism in treating DMCI have been preliminarily investigated (Fang et al., 2009; Fang et al., 2012a). However, the mechanism of FYXN in angiogenesis has not been fully elucidated yet.

Recent evidence has showed that Rab1 protein participates in revascularization by mediating angiotensin II type 1 receptor (AT1R) vesicle transport. Angiotensin II (AngII) may upregulate the vascular endothelial growth factor (VEGF) expression in ischemic tissues via the AT1R signal transduction pathway to promote angiogenesis (Forrester et al., 2018). The function of AT1R is closely related to intracellular transport. In the endoplasmic reticulum (ER), after being synthesized, folded and assembled, AT1R is transported to Golgi to fulfill post-translational modification, and then to the surface of cell-membrane by AngII to inspire the signal transduction pathway (Tadevosyan et al., 2017). Finally, AT1R degrades in lysosomes and can be reused. Current research (Lima et al., 2019) suggests that Rab1 can mediate protein transport from the ER to the Golgi apparatus (Batoko et al., 2000), and even regulate vascular endothelial cells like AT1R (Yin et al., 2011). Therefore, we speculated that Rab1 protein could mediate AT1R vesicle trafficking, which might play an important role in the angiogenesis in DMCI.

In this study, we sought to investigate the mechanism of FYXN in promoting angiogenesis in DMCI.

## Materials and Methods

### Experiments *in Vivo*

#### Preparation of Fuyuan Xingnao Decoction

**FIGURE 1:**
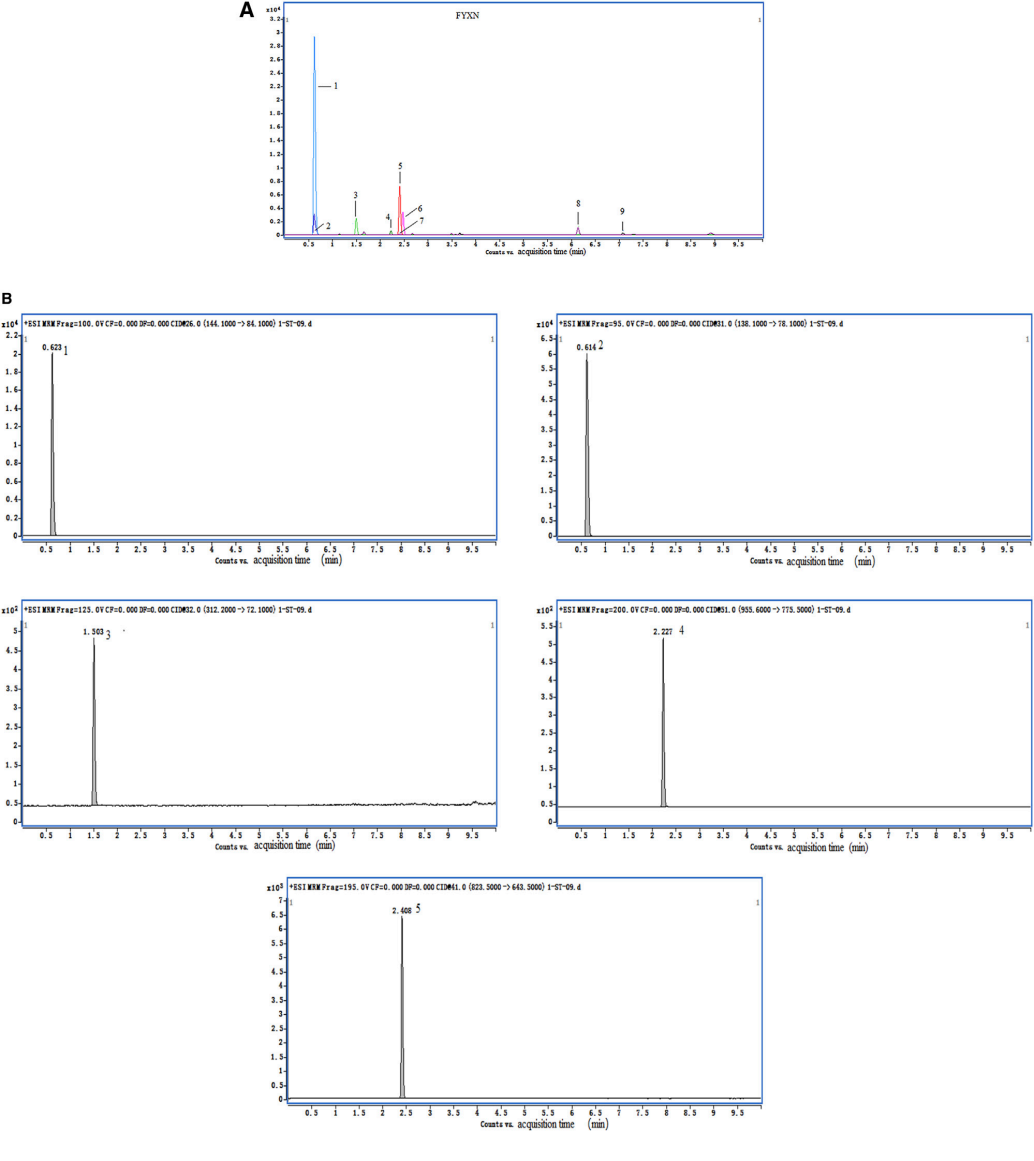
HPLC data of FYXN. **(A)** and **(B)** show total ion chromatograms and standard sample. The peaks indicate the presence of stachydrine hydrochloride (1), trigonelline (2), leonurine hydrochloride (3), notoginsenoside R1 (4) and ginsenoside Rg1 (5), which confirms the authenticity and major pharmacologically active constituents of FYXN.

FYXN, prepared from seven dried raw materials (Table 1) from the Pharmaceutical Department of Longhua Hospital, Shanghai University of TCM (Shanghai, China), was authenticated by a pharmacist of Longhua Hospital. The ingredients of FYXN, including Panax Ginseng C. A. Mey.(RS), Arisaema consanguineum Schott (TNX), Rheum officinale Baill (DH), Acorus gramineus Aiton (SCP), Panax Notoginseng (Burk.) F. H. Chen Ex C. Chow (SQ), Leonurus heterophyllus Sweet (YMC), leech (SZ) (https://pubmed.ncbi.nlm.nih.gov/30543914/), were mixed at a ratio of 10:15:12:10:10:30:10 (dry weight). The composition of FYXN was confirmed by high performance liquid chromatography (HPLC) (Figure 1).

**TABLE 1:** Herbs in FYXN.

Chinse name	Scientific name		Dosage (g)	Origin (China)
Ren Shen	Panax Ginseng C. A. Mey	Ginseng	10	Heilongjiang
Tian Nan Xin	Arisaema amurense Maxim.; Arisaema erubescens (Wall.) Schott; Arisaema heterophyllum Blume	Pinellia pedatisecta, arisaema heterophyllum, arisaema amurense	15	Sichuan
Da Huang	Rheum officinale Baill.; Rheum palmatum L.; Rheum tanguticum (Maxim. ex Regel) Balf.	Rheum palmatum, rheum tanguticum	10	Sichuan
Shi Chang Pu	Acorus gramineus Aiton	Acorus gramineus	12	Hunan
San Qi	Panax notoginseng (Burkill) F.H.Chen	Sanchi	10	Yunnan
Yi Mu Cao	Leonurus sibiricus L.	Leonurus sibiricus	30	Hubei
Shui Zhi	Whitmania pigra Whitman	Leech	10	Shandong

#### Experimental Animals

All animal experiments were approved by the Experimental Animal Administration Committee of Shanghai University of TCM. Male Sprague Dawley (SD) rats (250 ± 25 g) were purchased from Vital River Co. (Beijing, China; certification number: SCXK (Jing): 2012–0001). The rats were caged under specific pathogen-free (SPF) conditions at a temperature of 23.5 ± 1.5°C with a 12 h light/12 h dark cycle. They had free access to water and standard rodent food. All surgeries were performed under anesthesia induced by 2% sodium pentobarbital. Efforts were made to minimize animal suffering during the experiments.

Streptozotocin (STZ) injection: The DM animal model was established based on high-fat and high-sugar diet and STZ injection according to the previous literature. The injection was completed within 30 min. Before and after STZ injection, the body weight and blood sugar of all rats were recorded.

The preparation of permanent middle cerebral artery occlusion (MCAO): The rat was placed in a supine position after anesthesia. A midline incision was made in the neck. The right common carotid artery (CCA), external carotid artery (ECA) and internal carotid artery (ICA) were carefully isolated. Two microaneurysm clips were placed at the CCA and ECA to prevent bleeding during insertion of the suture. A hole was then made on the CCA between the clips using the needle of 1 ml injector. A 34 mm silica gel thread (Beijing Cinontch Co. Ltd., Beijing, China) was inserted into the internal carotid artery through a small incision on the right CCA and advanced by 18 ± 2 mm to occlude the middle cerebral artery until a mild resistance was felt. The vessel clips were then withdrawn. Rats of sham group underwent all the above surgical procedures, except for artery occlusion. Subsequently, the thread was left in place after occlusion until sacrifice. The animals were maintained at 32°C–35°C using a heating pad (Beijing Cinontch Co. Ltd., Beijing, China).

#### Grouping and Treatment

A total of 150 rats were randomized into five groups (30 in each group): 1) DM sham group: with induced-DM, receiving sham surgery and the same volume of saline as FYXN in FYXN group; 2) DMCI group: with induced-DM, receiving MCAO and the same volume of saline as FYXN in FYXN group; 3) DMCI + FYXN group: with induced-DM, receiving MCAO and FYXN (10.4 g/kg); 4) DMCI + Nim & Met group: with induced-DM, receiving MCAO and metformin (27 mg/kg) and nimodipine (2.16 mg/kg); 5) Normal control (NC) group: without induced-DM and receiving the same treatments as DM sham group. All the rats were treated twice a day for 7 days.

#### Measurement of Blood Glucose

After administration of high fat diet and STZ injection, the blood was sampled from the tail vein for blood glucose measurement to ensure the successful establishment of DM model. The blood glucose was also observed before and seven days after MCAO.

#### Assessment of Neurological Function

At day 1 and 7 after MCAO, neurobehavioral functions of the rats were evaluated by Bederson scale (Bederson et al., 1986): 0, no neurological deficits; 1, mild deficits (failure to fully extend the right forepaw); 2, moderate deficits (circling to the right); 3, moderate deficits (falling to the right); 4, severe deficits (unable to walk spontaneously with depressed levels of consciousness). The rats scored 0 were excluded, and those with score ≥1 were enrolled. Higher neurological deficit scores indicated more severe impairment caused by motor motion injury.

#### Infarct Volume Quantification

The fresh brain tissues were frozen for 30 min at −20°C and sliced into cross-sections of 2.0 mm. The slices were incubated in 2% solution of 2,3,5-TTC (Sigma, USA) at 37°C for 30 min (in dark), then fixed in 4% paraformaldehyde overnight at 4°C. The normal brain tissues appeared red, and infarcted tissues were pale. The sample sections were photographed and the infarct volumes were calculated by ImageJ.

#### Hematoxylin-Eosin Staining and Immunohistochemical Assay

Hematoxylin-eosin (HE) staining was used to detect the pathological changes in the ischemic brain tissues of DMCI rats. The brain tissues embedded in paraffin were cut into 5 μm slices, dewaxed in xylene and dehydrated by fractional alcohols. Afterward, the sections were stained with hematoxylin. Consequently, the vascular morphology of the ischemic brain tissues was examined using an Olympus, IX-71 microscope (Olympus, Japan).

For immunohistochemical analysis, the brain samples were extracted and fixed in 4% paraformaldehyde for 24 h. Then paraffin-embedded sections (5 μm) were mounted on glass slides, mixed with 3% H_2_O_2_ for 10 min to quench endogenous peroxidase activity, and blocked. Then, they were incubated at 4°C overnight with the primary CD31 (1:500 dilution; Abcam), washed by phosphate buffer saline (PBS), and incubated with second antibody horseradish peroxidase-conjugated at 37°C for 50 min. After the subsequent DAB incubation and visualization, the images were detected under microscope using the Image J analysis system.

#### Transmission Electron Microscope Examination

The cerebral cortex tissues were immediately fixed by immersion in 2% glutaraldehyde solution buffered with 0.2 M cacodylate buffer and post-fixed in osmium tetroxide before embedded in epoxy resin. Ultra-thin sections stained with uranyl acetate and lead citrate were examined. The ultrastructure of rat brain microvasculature was observed and imaged using a JEM-2100 transmission electron microscope.

#### Real-Time Fluorescence Quantitative Real-Time Polymerase Chain Reaction

Total RNA was isolated and then reversely transcribed to cDNA. RT-qPCR was conducted using an ABI Step One Plus Real-time PCR System (Applied Biosystems, USA) with fluorescent dye (SYBR Green I, Takara) as follows: 50°C for 2 min, followed by 95°C for 10 min, then 40 cycles at 95°C for 15 s followed by 60°C for 1 min. The relative mRNA expression was normalized to GAPDH level and analyzed using the 2^−ΔΔCT^ method. The primers (Table 2) were synthesized by Sangon Biotechnology Co. (Shanghai, China).

**TABLE 2:** Sequences of primers used *in vivo* and vitro experiments.

Name	Forward primer (5′–3′)	Reverse primer (5′–3′)
Rab1A	GCT​ACG​CCA​GCG​AAA​ATG​TC	AAA​AGG​ACG​GAG​GCG​GAT​TT
Rab1B	CCA​CCA​TCT​TGG​AAC​GGG​A	CCT​AGG​AGA​GGG​AGG​CAC​TT
AT1R	CTC​TGC​CAC​ATT​CCC​TGA​GTT​A	TGG​GGC​AGT​CAT​CTT​GGA​TTC
AngII	GTC​GGT​TTT​TGT​GCT​GGG​TC	GCA​AGC​CAT​TTT​CAC​AGG​CA
VEGF	TGG​ACC​CTG​GCT​TTA​CTG​CT	AGG​CTC​ACA​GTG​AAC​GCT​CC
GAPDH	AGT​GCC​AGC​CTC​GTC​TCA​TA	GAT​GGT​GAT​GGG​TTT​CCC​GT
A-1	TCC​AGC​ATG​AAT​CCC​GAA​T	TCC​ACG​TAA​CTC​CGA​GAA​T
A-2	TCT​CCT​TAG​GTT​TGC​GGA​T	TCTATTTGGGTTGCCCGA
A-3	GCT​AAG​AAC​GCA​ACG​AAT​G	CTCAAGAACGAGCAAATG
B-1	GCA​CCC​ACG​TTA​TCT​TCT​T	GCA​ACG​CTT​TAT​CTC​CCT​T
B-2	GCG​TCT​GTA​ATG​GCA​GTA​A	GCG​TGT​TAA​CGG​GAT​CTA​A
B-3	GGT​ATC​ACC​TGC​CTT​TCT​T	GGT​CAC​GTC​TCC​TTA​TCT​T

#### Western Blotting

Protein was separated by 10% sodium dodecyl sulfate polyacrylamide gels (10% SDS-PAGE), and electrophoretically transferred to PVDF membranes (Millipore). After blocking, the protein was stained overnight at 4 °C respectively with primary GAPDH (diluted 1:5,000; Proteintech-USA), AT1R, AngII, Rab1a, Rab1b and VEGF (diluted 1:1,000; Proteintech-USA). The blots were then washed with TBST and incubated for 1 h with secondary antibodies conjugated to horseradish peroxidase (diluted 1: 10,000; Beyotime-Shanghai). The protein bands were scanned using Quantity One 4.31 software (bio-rad, USA).

### Experiments *in Vitro*

#### Preparation of Drug Serum

Twenty healthy female SD rats (Laboratory Animal Center of SHUTCM) were randomized into two groups: blank-controlled trial group and FYXN group, which were treated with normal saline (1 ml/100 g·body weight) or FYXN (10.4 g/kg) respectively, twice a day by oral gavage. Rats were sacrificed on the fourth day at 2 h after the last oral administration. Blood samples were collected through abdominal aorta and centrifuged. Supernatants were filtered and sterilized in 56°C water for 30 min, and stored at 4°C. The serum was added into the medium directly as the drug.

#### Primary Brain Microvascular Endothelial Cells Isolation and Culture

Sprague Dawley rats (250 ± 25 g, male) were purchased from the Vital River Co. (Beijing, China; certification number: SCXK (Jing): 2012–0001). After the successful establishment of the DMCI model, only the cerebral cortex was preserved and placed in Dulbecco’s modified Eagle’s medium (DMEM). The BMECs were isolated by suspension and centrifugation. DMEM containing 20% fetal bovine serum (FBS, Hyclone, USA) and 100 μg/ml heparin sodium was added to the purified microvessel cells, which were then inoculated onto a 35 mm Petri dish coated with rat tail collagen.

#### Brain Microvascular Endothelial Cells Transfection Rab1 Lentivirus

The purpose of lentivirus transfection was to reduce the expression of Rab1 by interfering with Rab1 gene. Lentiviral vectors used in this study were constructed by Jikai Genochemical Technology Co., Ltd. (Shanghai, China). The two subtypes of Rab1 (Rab1a and Rab1b) were interfered respectively. The Rab1a-siRNA sequence and Rab1b-siRNA sequence used were shown in Table 2.

According to the above sequence, the PEI virus was packaged and transfected with PEI according to the instruction. The transfection complex of PEI/DNA mixture cultured with 293 T cell for 48–72 h to collect the produced virus, which was then added to the 6-well plates. The fluid was changed according to the BMECs status 24 h later.

#### Cell Proliferation Assay

The transfected cells were seeded at a density of 1 × 10^4^ cells/well in 96-well flat bottom plates, cultured for 24 h, then treated with 10% FYXN serum or FBS for 24 h. The proliferation assay was performed 6 h following the addition of BMECs reagent (10 ng/ml) using Cell Proliferation Assay Kit (Beyotime, Shanghai). The absorbance values measured at 492 nm wavelength represented the rate of proliferating cells.

#### Cell Migration Assay

For migration assay, the transfected cells were cultured with FYXN serum or FBS for 24 h and starved for 2 h before the experiment. The suspension (200 μL) was added into the upper chambers, and 700 μL of supplemented DMEM was added into the lower chambers. After a 24 h culture, the cells having migrated to the lower chambers were treated with methanol for 3 min, then stained by 0.1% crystal violet for 20 min. Stained cells were observed under an inverted microscope, and five randomized fields were selected for cell count.

#### Western Blot Analysis

As described in 2.1.10.

#### Immunofluorescent Staining

Immunofluorescence staining was used to detect the AT1R expression on the cell membrane and endoplasmic reticulum of cerebral microtubules. Cerebral cortex was fixed by cold methanol and then permeabilized in PBS. The sections were first incubated with 10% goat serum and then with primary AT1R overnight at 4°C, followed by incubation with second anti-mouse antibodies (1:100 dilution, Beyotime). After washing the slides with PBS for three times, the nuclei were stained with DAPI for 5 min at room temperature (RT). Then 2 μL of anti-fluorescence quench solution was added to cover the cell slide, inverted and observed under the fluorescence microscope.

#### Statistical Analysis

All experimental data were tested for normality before statistical analysis. The data consistent with normal distribution were described as mean ± standard deviation. One-way ANOVA was used to test the overall difference and Bonferroni method was used for pair comparison. The data that did not conform to the normal distribution were described by median M(IQR), and statistical analysis was conducted by using multiple local rank sum test (Kruskal Wallis rank sum test).

## Results

### *In Vivo* Experiments

#### Fuyuan Xingnao Decoction Decreased Blood Glucose in Diabetes Mellitus Complicated with Cerebral Infarction Rat Model

**FIGURE 2:**
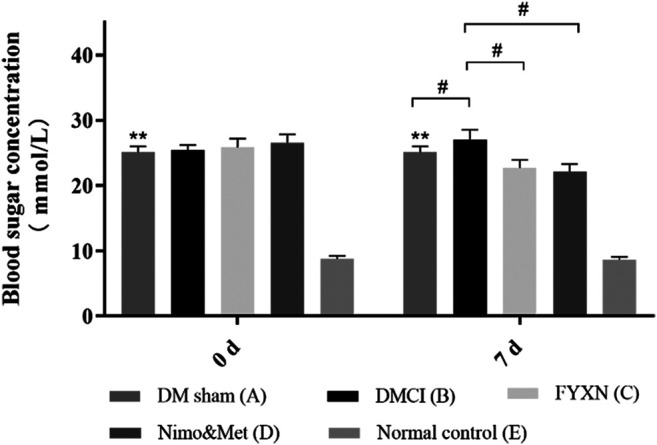
FYXN decreased blood glucose in a rat DMCI model (mean ± SD, mmol/L), *p < 0.05, **p < 0.01 compared with NC group; #*p* < 0.05 compared with DMCI group.

Figure 2 presented the blood glucose of rats in different groups. Compared with NC group, the blood glucose of rats in the other four groups was significantly higher (*p* < 0.01), indicating the successful establishment of DM rat model and ensuring the reliability of the subsequent experiment. Before dosing, compared with DM sham group, the blood glucose increased in DMCI, FYXN and Nim & Met groups, but no significant difference was found (*p* > 0.05). After 7 days of DMCI treatment, the blood glucose in FYXN group and Nim & Met group decreased dramatically, compared with DMCI group (*p* < 0.05).

#### Fuyuan Xingnao Decoction Treatment Improved Neurological Deficit in Diabetes Mellitus Complicated with Cerebral Infarction Rats

**FIGURE 3:**
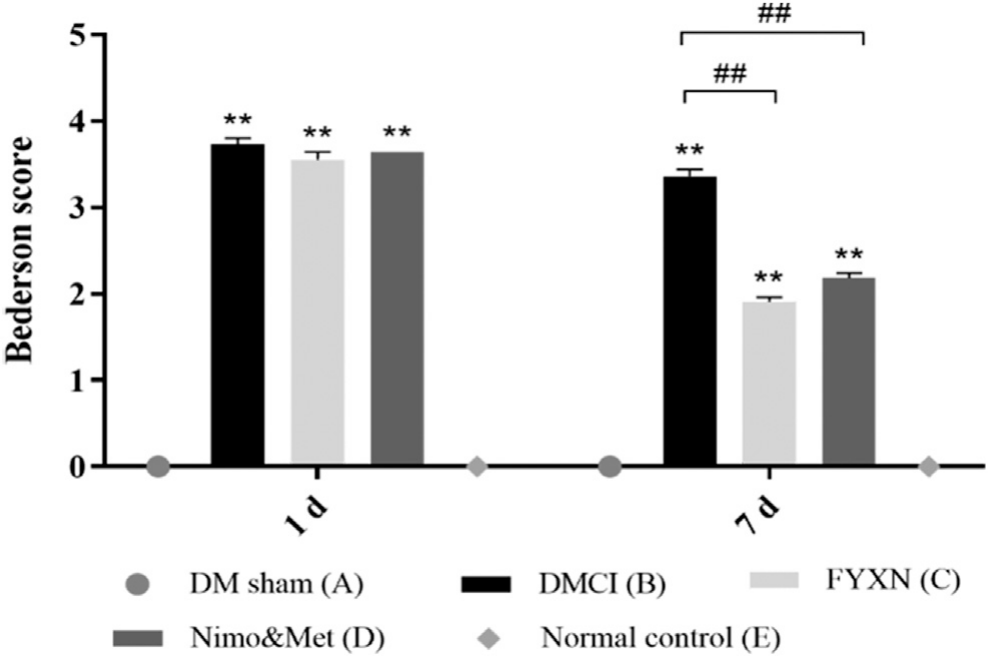
Neurological functional results. Data of neurological deficit scores are shown as mean ± SD in each group. *p < 0.05, **p < 0.01 compared with NC group; #*p* < 0.05, ##*p* < 0.01 compared with DMCI group.

The neurological function of all rats was assessed before treatment and at day 7 after treatment. Rats in DM sham group and NC group showed no behavioral impairments, while significant difference was found when compared with DMCI group, which confirmed the successful establishment of animal model. No significant difference in neurological deficit scores was found among DMCI group, FYXN group and Nim & Met groups before FYXN administration (*p* > 0.05, Figure 3). After a 7 days treatment, compared with DMCI group, the neurological scores were significantly decreased in FYXN group and Nim & Met groups (*p* < 0.01). These results demonstrated that FYXN exerted neuroprotective effects against neurological deficits.

#### Fuyuan Xingnao Decoction Attenuated Cerebral Infarct in Diabetes Mellitus Complicated with Cerebral Infarction Rats

**FIGURE 4:**
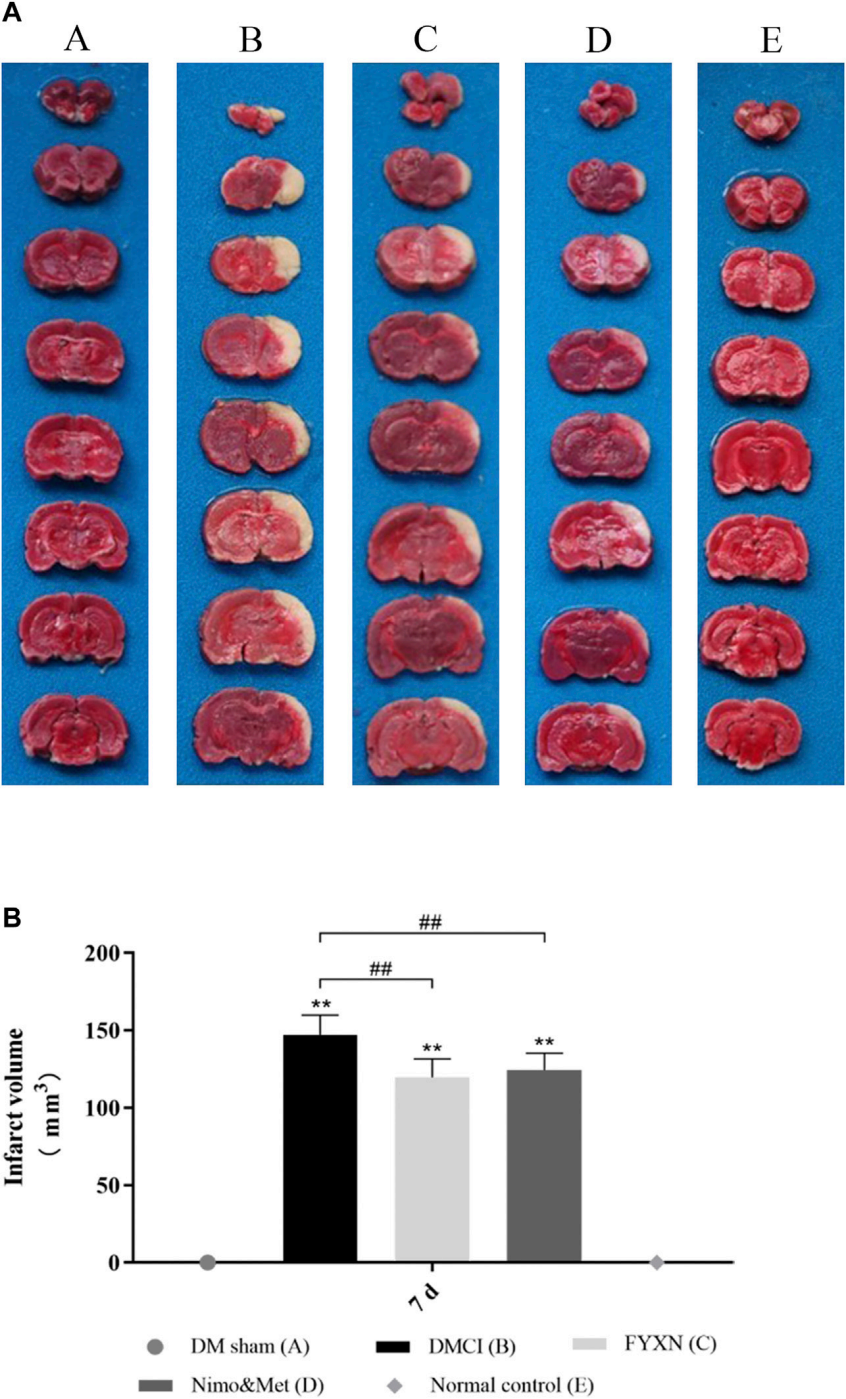
**(A)** Focal cerebral infarct volume in the experimental groups after 7 days of ischemia followed by 7 days of treatment; 2,3,5-Triphenyltetrazolium chloride staining shows non-infarct (red) and infarct (white) regions. **(B)** Data of cerebral infarct volume are shown as mean ± SD in each group. *p < 0.05, **p < 0.01 compared with NC group; #*p* < 0.05, ##*p* < 0.01 compared with DMCI group.

TTC staining was conducted to assess CI. The rats developed prominent CI after MCAO operation (*p* < 0.05; vs. DM sham group and NC group; Figures 4AB). The CI areas were significantly decreased in FYXN and Nim & Met groups compared with those in DMCI group at day 7 after treatment (*p* < 0.05 in both groups; Figures 4A,B). However, the cerebral infarct areas between FYXN and Nim & Met groups exhibited no significant difference (*p* > 0.05).

#### Fuyuan Xingnao Decoction Caused Morphological Changes and Increased Cerebral Microvasculature Density

HE staining was performed to evaluate the effect of FYXN on ischemic brain tissues *in vivo*. Neurons and few normally arranged gliocytes were observed in DM sham and NC groups; spongy brain tissues, severe edema, wide vacuolization, macrophage infiltration, disordered and necrotic neurons in DMCI group; mild edema, local necrosis, aggregation of gliocytes, local vacuolization, and glia in FYXN and Nim & Met groups.

**FIGURE 5:**
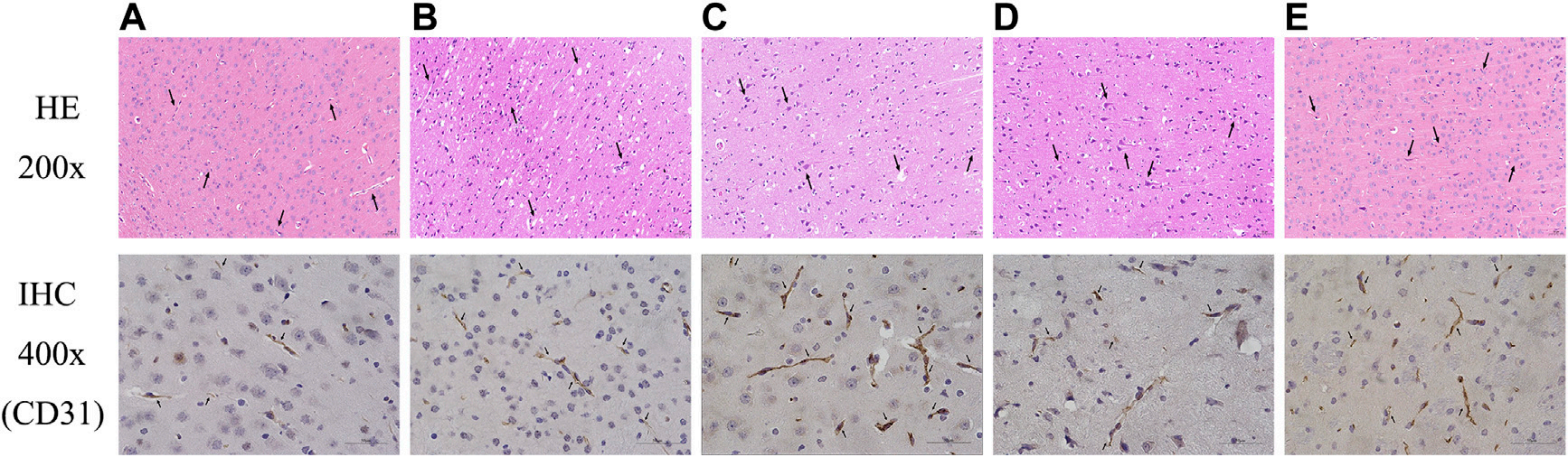
FYXN improved morphology and promoted angiogenesis in DMCI rats. Representative images of HE and immunohistochemistry are shown as follows: **(A)** DM sham group, **(B)** DMCI group, **(C)** FYXN group, **(D)** Nim & Met group and **(E)** NC group.

To further analyze the effects of FYXN on angiogenesis *in vivo*, we examined the expression of CD31, an angiogenesis marker in brain tissues, by immunohistochemistry. We found that FYXN treatment significantly increased the density of CD31 staining in the cortical areas (Figure 5). These results suggested that FYXN increased the development of cerebral microvasculature.

#### Fuyuan Xingnao Decoction Altered Postischemic Morphology and Ultrastructure of Cerebral Microvessels

**FIGURE 6:**
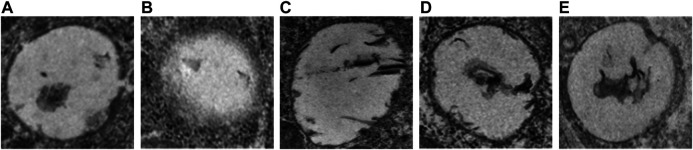
TEM micrograms. Representative cerebral microvascular ultrastructures (x 10,000). **(A)** DM sham group, **(B)** DMCI group, **(C)** FYXN group, **(D)** Nim & Met group and **(E)** NC group. An intact cerebral microvascular ultrastructure is shown in DM sham and NC groups, and a disordered cerebral microvascular ultrastructure is illustrated in DMCI, FYXN and Nim & Met groups. Scale bars = 200 nm.

The DMCI group showed segmental unclear even stenosis, defects and luminal reduction in the basement membrane structure under transmission electron microscope (TEM). An intact cerebral microvascular ultrastructure was shown in DM sham and NC groups. A disorderly cerebral microvascular ultrastructure was observed in DMCI, FYXN group and Nim & Met groups, indicating significant improvement. However, the integrity of cerebral microvessels in FYXN and Nim & Met groups was improved after treatment. Edema and architectural disruption of cerebral microvessels were not observed in DM sham and the NC groups, but basement membrane thickening was found in DM sham group (Figure 6).

#### Fuyuan Xingnao Decoction Treatment Increased the Expressions of AT1R, Ang II, Rab1a, Rab1b and Vascular Endothelial Growth Factor mRNA in Ischemic Cerebral Cortex Tissues

**FIGURE 7:**
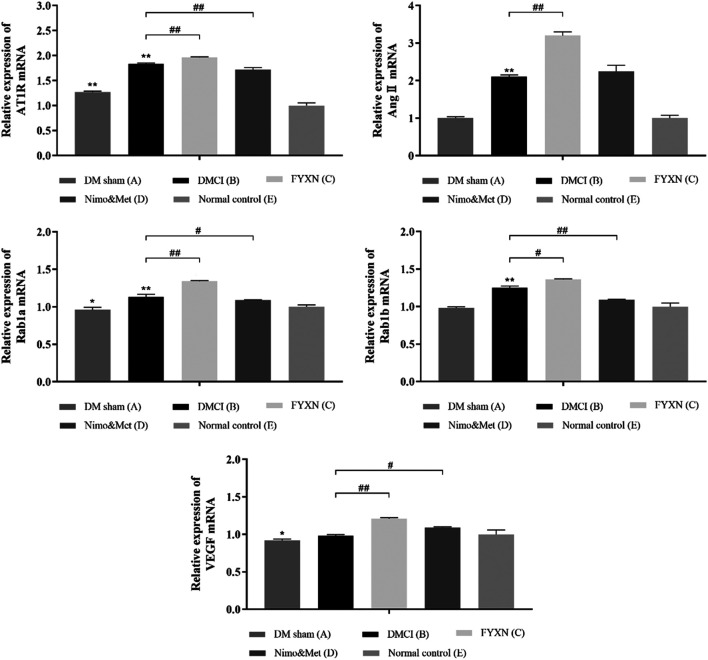
Effects of FYXN treatment on the mRNA levels of AT1R, Ang II, Rab1a, Rab1b and VEGF in ischemic cerebral cortex tissues. Expression levels were detected by RT-qPCR. *p < 0.05, **p < 0.01 compared with NC group; #*p* < 0.05, ##*p* < 0.01 compared with DMCI group.

RT-qPCR results showed the expressions of AngII and Rab1b mRNA were not significantly different between DM sham and NC groups (*p* > 0.05). Compared with NC group, the expressions of AT1R, Ang II, Rab1a and Rab1b mRNA were increased in DMCI, FYXN and Nim & Met groups (*p* < 0.01). Compared with DMCI and NC groups, the mRNA levels of AngII, AT1R, Rab1a, Rab1b and VEGF were significantly higher in FYXN group (*p* < 0.01); meanwhile, the mRNA levels of AT1R and Rab1b, Rab1a and VEGF were significantly increased in Nim & Met group (*p* < 0.05). However, the mRNA level of AngII showed no significant difference between DMCI and Nim & Met groups (Figure 7).

#### Fuyuan Xingnao Decoction Activated the Rab1/AT1R Pathway

**FIGURE 8:**
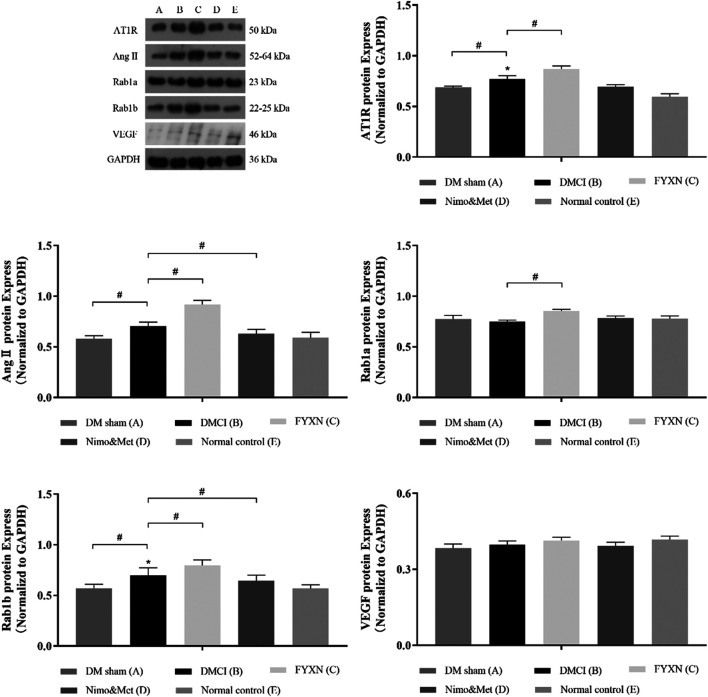
Effects of FYXN treatment on the expression of AT1R, Ang II, Rab1a, Rab1b and VEGF protein in ischemic cerebral cortex tissue. Their expressions were detected by Western Blotting. *p < 0.05 compared with NC group; #*p* < 0.05 compared with DMCI group.

Western blotting was used to evaluate the expression levels of AT1R, Ang II, Rab1a, Rab1b and VEGF in protein extracts from cerebral cortex tissues. It was revealed that the levels of AT1R, Ang II and Rab1b in the cerebral cortex tissues were remarkably increased in DMCI group, compared to DM sham group and NC group (*p* < 0.05). However, the levels of Rab1a and VEGF were reduced (*p* > 0.05). At the same time, the levels of AT1R, Ang II, Rab1a and Rab1b were significantly upregulated in FYXN group than in DMCI group (*p* < 0.05). Compared with DMCI group, the levels of AngII and Rab1b were reduced (*p* < 0.05), Rab1a upregulated (*p* > 0.05), AT1R and VEGF almost unchanged (*p* > 0.05) in Nim & Met group. These findings indicated that the Rab1/AT1R signaling pathway was involved in FYXN-induced angiogenesis *in vivo*. As expected, no significant difference was observed between DM sham group and NC group (Figure 8).

### *In Vitro* Experiments

#### Brain Microvascular Endothelial Cells Culture and Status Map of Virus Infection

**FIGURE 9:**
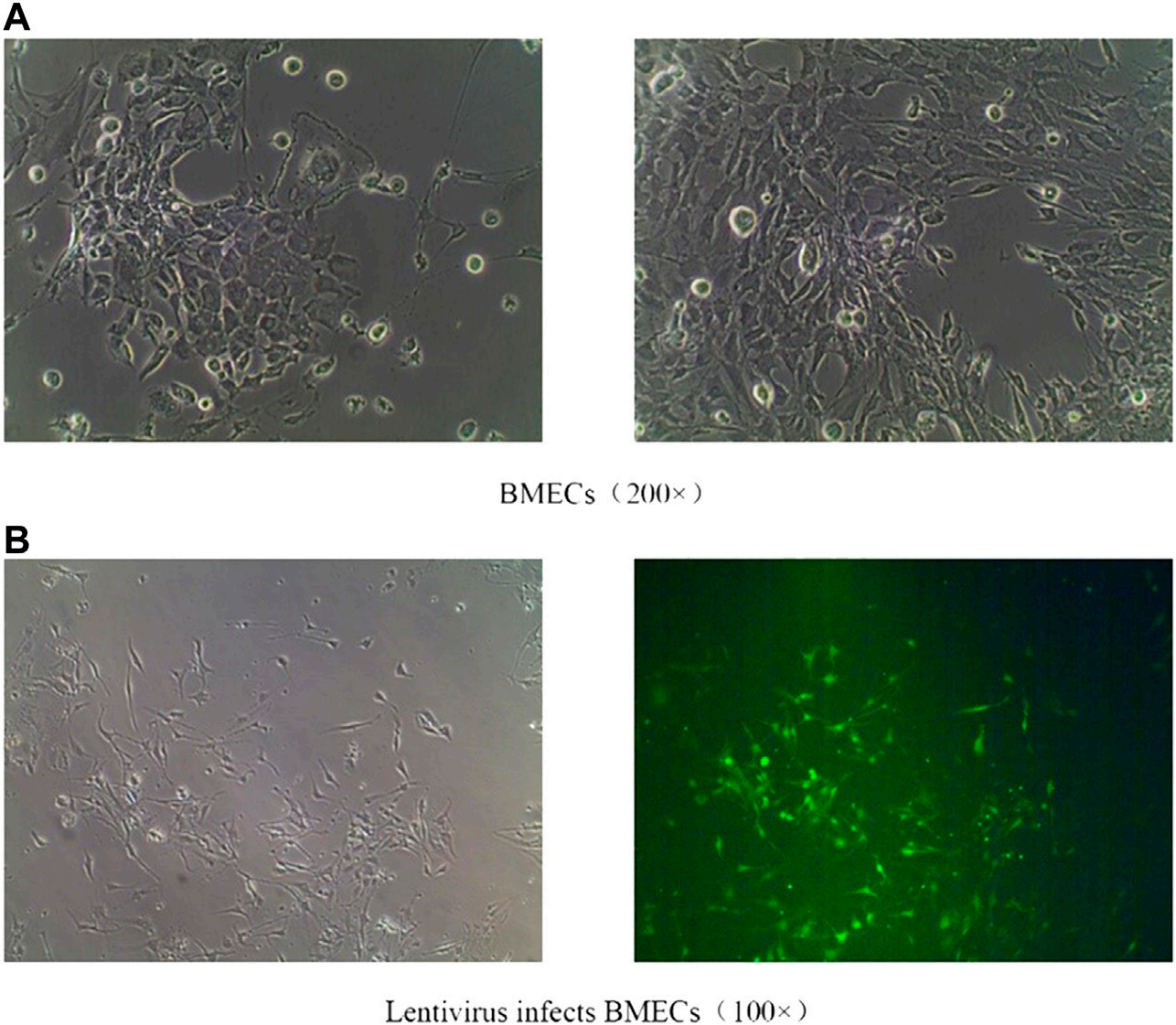
**(A)**: Normal BMECs; **(B)**: Effective transfection of BMECs with lentiviruses.

After isolation, the cultured BMECs showed irregular fusiform and long processes (Figure 9A). Recombinant lentivirus packaged Rab1-WT or siRNA was constructed, transfected into BMECs, and treated with FYXN to explore the angiogenesis-associated pathway. The efficiency of lentivirus transfection wad indicated with green fluorescence. As shown in (Figure 9B), the transfection efficiency was 80%.

#### Fuyuan Xingnao Decoction Enhanced the Proliferation of Brain Microvascular Endothelial Cells

**FIGURE 10:**
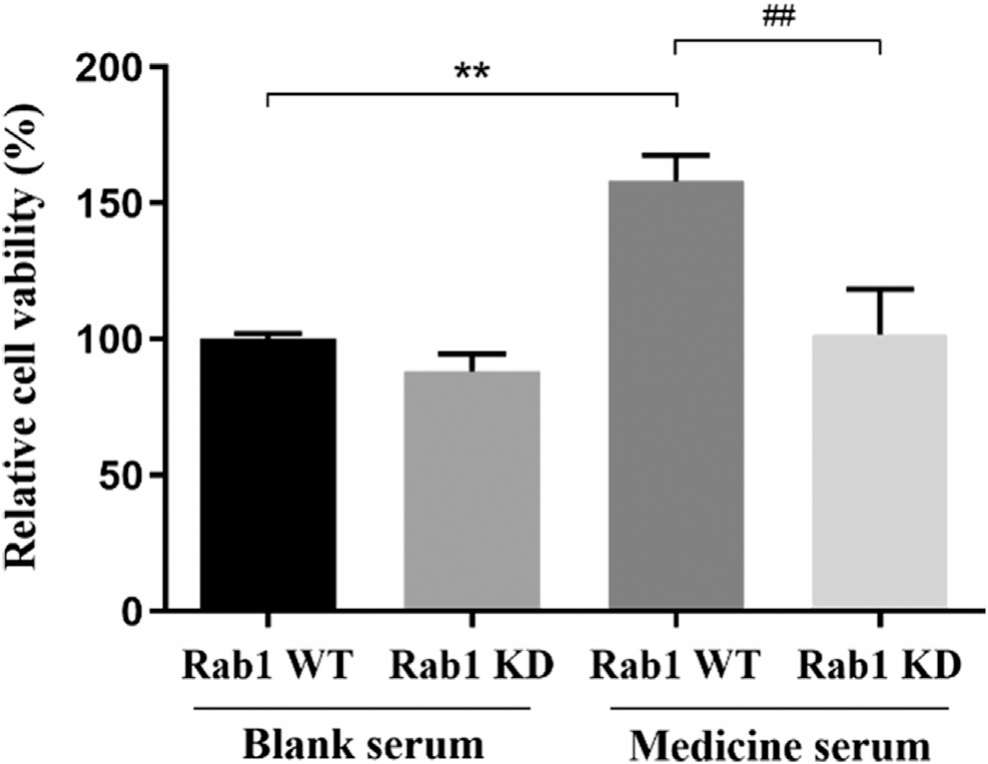
Effect of FYXN serum on the proliferation of BMECs, **p < 0.01 compared with NC + Rab1WT group; ##*p* < 0.01 compared with the FYXN + Rab1WT group.

The proliferation of BMECs was detected by MTT to evaluate the effect of lentivirus transfection and the mechanism of FYXN in promoting BMEC proliferation. After transfection with lentivirus Rab1 WT and Rab1 siRNA for 24 h, the cell proliferation in FYXN + Rab1WT group was enhanced significantly, compared with NC + Rab1WT group (*p* < 0.001), and inhibited in FYXN + Rab1KD group, suggesting that FYXN might mediate the proliferation of BMECs through Rab1 (Figure 10).

#### Fuyuan Xingnao Decoction Promoted the Migration of Brain Microvascular Endothelial Cells

**FIGURE 11:**
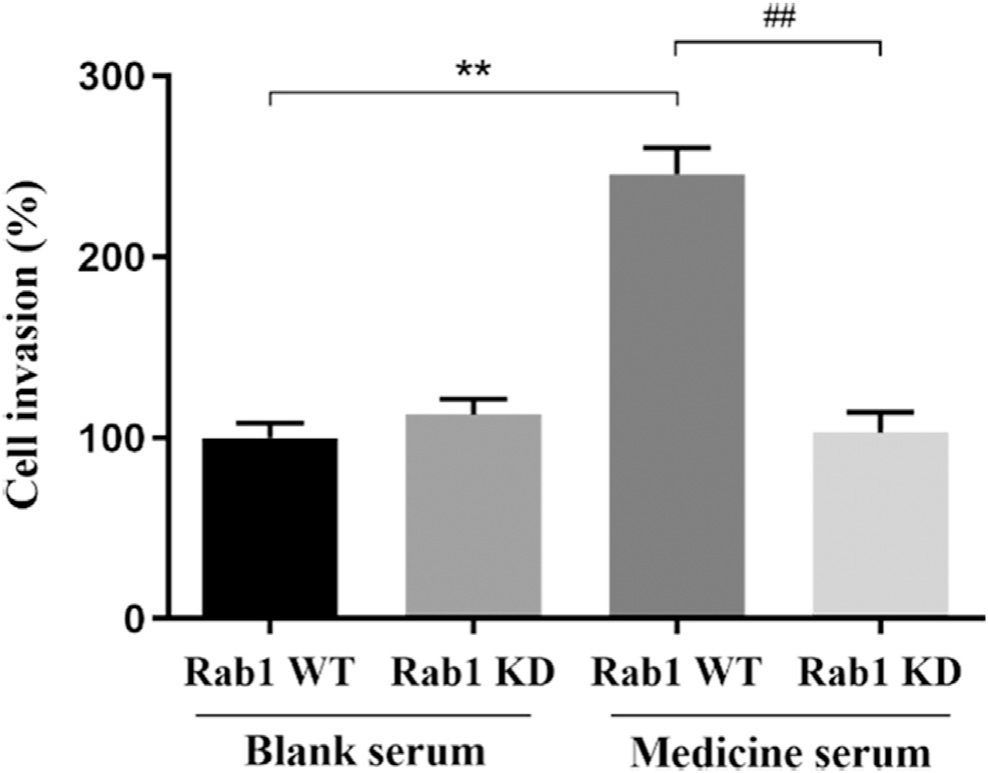
The effect of FYXN serum on the migration of BMECs, **p < 0.01, compared with NC + Rab1WT group; ##*p* < 0.01, compared with FYXN + Rab1WT group.

After 24 h of lentivirus Rab1-WT and Rab1-siRNA transfection, the migration of BMECs was promoted in FYXN + Rab1WT group, compared with NC + Rab1WT group (*p* < 0.001), but inhibited in FYXN + Rab1KD group, indicating that FYXN influenced the migration of BMECs through Rab1 (Figure 11).

#### Fuyuan Xingnao Decoction Upregulated the Expression of Angiogenesis-Associated Protein of Brain Microvascular Endothelial Cells

**FIGURE 12:**
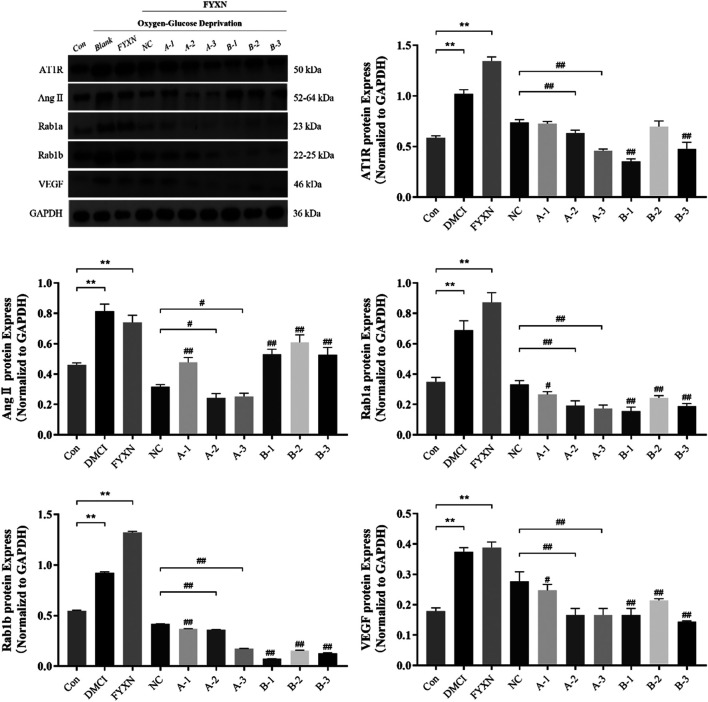
The expression levels of angiogenesis-associated proteins (AT1R, Ang II, Rab1a, Rab1b, VEGF) after interference with Rab1a and Rab1b *in vitro*. The Rab1a siRNA sequences of A1, A2 and A3, and the Rab1b siRNA sequences of B1, B2 and B3. *p < 0.05, **p < 0.01, compared with control group; #*p* < 0.05, ##*p* < 0.01, compared with NC group.

Results showed that the expression levels of AT1R, Ang II, Rab1a, Rab1b and VEGF in experimental groups were significantly higher than those in control group, and further increased after administration of FYXN drug serum (*p* < 0.05). Compared with NC group, the expression level of Rab1a decreased significantly after interference with A2 and A3 sequences (*p* < 0.05). After interference with Rab1a, the expression levels of AT1R, Ang II and VEGF were significantly lower than those in NC group (*p* < 0.05), suggesting that this decrease was caused by Rab1a. However, the expression levels of AT1R, Ang II and VEGF did not exhibit significant difference after interference with Rab1b (*p* > 0.05). The expression level of Rab1b was decreased after interference with Rab1b sequence (*p* < 0.05). Nevertheless, the expression levels of AT1R, Ang II and VEGF were not significantly decreased after interference with Rab1b, compared with those in NC group (*p* > 0.05). To sum up, the interference effect on Rab1 gene was stronger, and A2 and A3 sequences could regulate the expression levels of angiogenesis-associated proteins (Figure 12).

#### Effects of Fuyuan Xingnao Decoction on AT1R Expression on Brain Microvascular Endothelial Cells Membrane and Endoplasmic Reticulum

**FIGURE13:**
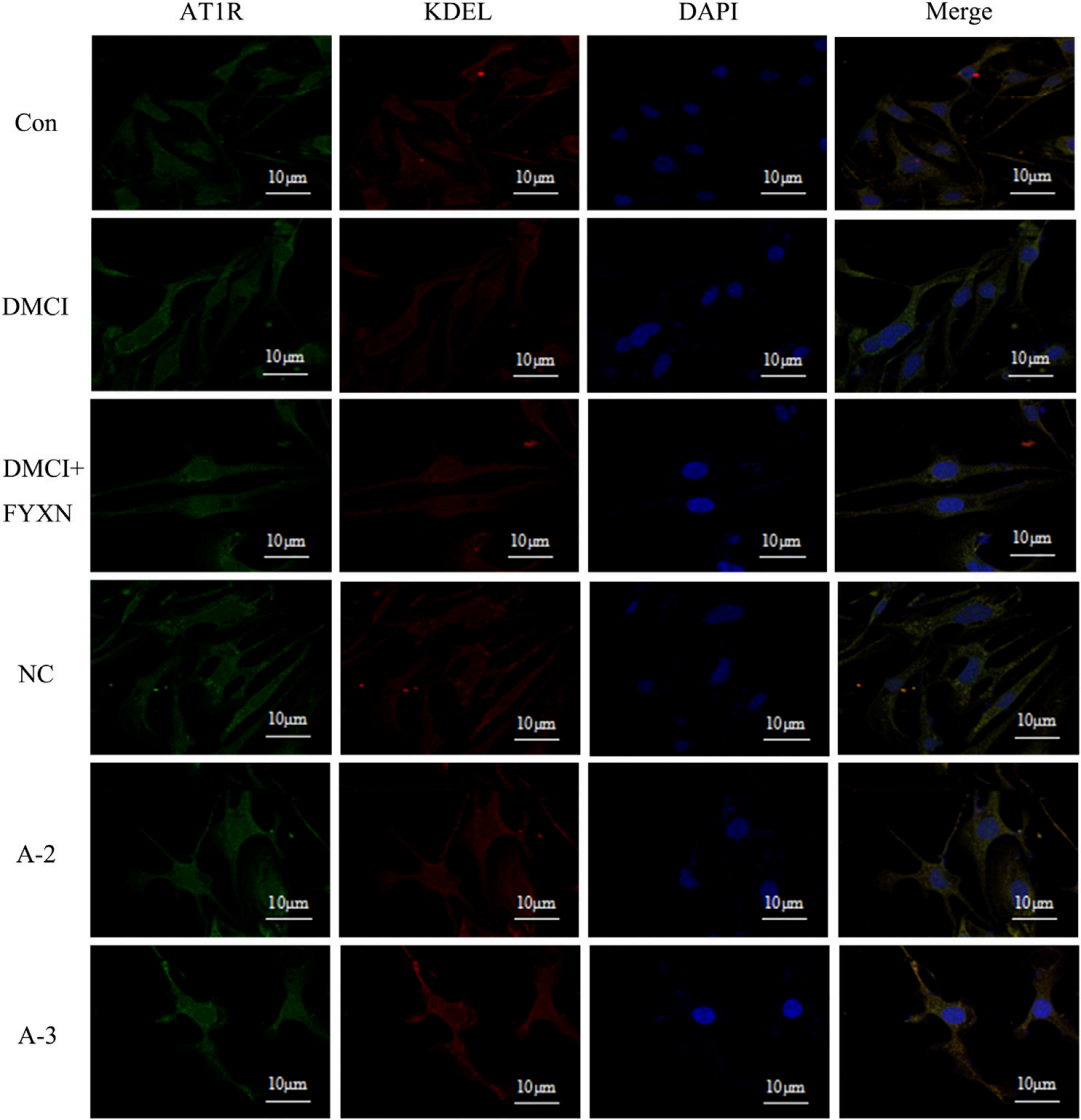
Immunofluorescence staining was used to detect the expression of AT1R on the membrane and endoplasmic reticulum in BMECs treated with FYXN serum. A2 and A3 are Rab1a siRNA sequences respectively.

Compared with control group, the fluorescence intensity of AT1R on the cell membrane in DMCI group slightly increased, but then dropped in DMCI + FYXN group. The fluorescence intensity of AT1R decreased slightly after Rab1a interference. AT1R expression did not change in endoplasmic reticulum in DMCI and DMCI + FYXN groups. The fluorescence intensity of AT1R in endoplasmic reticulum increased after Rab1a interference. In summary, AT1R was mainly expressed on the membrane of BMECs after Rab1 WT transfection, but on the endoplasmic reticulum after Rab1siRNA transfection (Figure 13).

## Discussion

FYXN contains a long list of active compounds effective for stroke, such as stachydrine hydrochloride (Miao et al., 2017), trigonelline (Di Lorenzo et al., 2019; Pravalika et al., 2019), leonurine hydrochloride, notoginsenoside R1 and ginsenoside Rg1 (Bai et al., 2018). Previous research has demonstrated that FYXN could lower serum glucose, improve insulin resistance and reduce neurologic deficits in DMCI patients (Fang et al., 2009; Fang et al., 2012b; Huang et al., 2009). FYXN can reduce cerebral infarction volume, facilitate brain microcirculation, normalized vascular morphology and density in the ischemic area in DMCI rats (Fang et al., 2010; Huang et al., 2013; Zhao et al., 2013). In addition, FYXN can upregulate the expression of cyclin E1 and CDC25A and downregulate the expression of microRNA-503 and miRNA-320, a mechanism that promoting the migration, proliferation and tube formation of BMECs *in vitro* (Shen et al., 2018; Shen et al., 2019a; Shen et al., 2019b). All these evidences supported the potential of FYXN in enhancing angiogenesis and vascular remodeling.

Multiple reports have shown that revascularization in the ischemic injury area of DMCI is vital to the recovery of neurological functions (Zhang et al., 2015; Rust et al., 2019). Efficient angiogenesis in ischemic areas could significantly improve the prognosis of DMCI (Ruan et al., 2015). However, the mechanism of angiogenesis in DMCI has not been fully elucidated. Evidence has shown that the vesicle transport of AT1R is mediated by Rab1 protein, which plays an important role in revascularization. Ang II, an octopeptide compound hormone (Lian et al., 2018; Santos et al., 2018), activates a variety of intracellular signal transduction pathways and exerts biological effects by binding to specific receptors on cell membrane (Liu et al., 2017). AT1R and AT2R, two subtypes of Ang II receptor, are members of the seven-time transmembrane G protein-coupled receptor superfamily (Preu et al., 2017). Ang II can not only increase the number of EPCs, but also enhance the proliferation, migration, adhesion and angiogenesis of EPCs *in vitro*. Ang II upregulates the expression of VEGF and other pro-angiogenic cytokines in early EPCs mediated by AT1R (Shahvegharasl et al., 2020), and promotes angiogenesis (Childers, 2015; Caillon et al., 2016). EPCs, a pool of circulating bone-marrow derived cells, mobilize after an ischemic injury into the damaged endothelium, to form new vessels, or secrete trophic factors stimulating vessel remodeling (Esquiva et al., 2018). Thus, there is a positive correlation between Ang II and VEGF in angiogenesis. It is suggested that Ang II may upregulate the expression of VEGF through AT1R signal transduction pathway to promote angiogenesis (Zhan et al., 2019).

The function of AT1R relies on intracellular transport (Liu et al., 2018). AT1R is synthesized on the endoplasmic reticulum and transported to Golgi apparatus for post-translational modification, then to Ang II on the cell membrane surface to stimulate signaling pathways. Finally, it binds to Ang II AT1R to degrade lysosomes (Vasile et al., 2020). In this process, vesicles transport neurotransmitters, proteins or other substances to specific cell sites (Sheng et al., 2018). In eukaryotic cells, the proteins, polynucleotides, polysaccharides and other biomolecules are transported through different types of vesicles. Rab proteins participate in vesicle transport through exocytosis and endocytosis. Current studies have shown that Rab1 protein can regulate the anterograde transport of AT1R from endoplasmic reticulum, through Golgi apparatus and to cell surface, thus promoting vascular remodeling (Connor et al., 2015; Raza et al., 2018; Vasile et al., 2020). Therefore, we primarily proved that AT1R vesicle transport might be mediated by Rab1 in vascular remodeling in DMCI (Schöppner et al., 2016; Yang et al., 2016). Our findings have brought new ideas and methods for the study on the pathogenesis and treatment of DMCI.

Hyperlipidemia is a vital risk for CI (Chen, B. et al., 2016). Acute hyperglycemia before or during local cerebral ischemia aggravates brain damage, which worsens the injury of brain tissue after cerebral infarction (Altintas et al., 2016; Liang et al., 2016). The infarct size of rats was reduced significantly in FYXN group and Nim & Met group, compared with DMCI group (*p* < 0.01), indicating that FYXN could improve the prognosis of DMCI. The results of IHC staining and TEM assay showed upregulated CD31, intact wall and increased diameter of blood vessels, suggesting that FYXN could promote angiogenesis. These results verified the effect of FYXN on vascular remodeling in DMCI, which lays a foundation for further studies on the molecular mechanism of DMCI pathogenesis. At mRNA and protein levels, we certified that FYXN enhanced the expressions of AT1R, Ang II, Rab1a, Rab1b and VEGF in ischemic cerebral cortex tissue using RT-qPCR and Western blotting. We speculated that FYXN could promote the angiogenesis in DMCI by activating the Rab1/AT1R pathway.

Our *in vitro* experiments further revealed that FYXN alleviated the cell injury by CI through upregulating the protein levels of AT1R, Ang II, Rab1a, Rab1b and VEGF. In addition, after interfering with Rab1, the expression levels of AT1R, Ang II and VEGF decreased, suggesting that Rab1 protein plays an important role in vascular remodeling in CI. The results of Western blot showed that upregulating the levels of AT1R, Ang II, Rab1a, Rab1b and VEGF could promote angiogenesis, improve blood supply and alleviate cerebral infarction in MCAO animals. In the diabetic group, the increase of cell membrane permeability might lead to the upregulation of AT1R under a long-term high glucose environment. The results of immunofluorescence staining showed that AT1R on the cell membrane of MCAO animals was higher than that in NC group. After FYXN administration, the expression of AT1R was further upregulated. After Rab1a silencing, the expression of AT1R on cell membrane decreased and that on endoplasmic reticulum increased. Thus, we speculated that Rab1a mediated the transport of AT1R from endoplasmic reticulum to cell membrane (Hernández-Socorro et al., 2017). Therefore, it is confirmed that FYXN can alleviate DMCI through promoting the proliferation and migration of BMECs to accelerate vascular remodeling.

In conclusion, FYXN can promote angiogenesis in DMCI rats through regulating Rab1/AT1R signal pathway. This study lays a foundation for the future study on the molecular mechanism of DMCI.

## Data Availability

The raw data supporting the conclusion of this article will be made available by the authors, without undue reservation.
